# Pearson Correlation in Determination of Quality of Current Transformers

**DOI:** 10.3390/s23052704

**Published:** 2023-03-01

**Authors:** Davorin Burgund, Srete Nikolovski, Dario Galić, Nedeljko Maravić

**Affiliations:** 1 Elektrogen d.o.o., 10000 Zagreb, Croatia; 2 Power Engineering Department, Faculty of Electrical Engineering, Computer Science and Information Technology, University of Osijek, 31000 Osijek, Croatia; 3 Faculty of Dental Medicine and Health, University of Osijek, 31000 Osijek, Croatia; 4 SFB Education Center for Technology and Management, Higher Technical School, 8953 Dietikon-Zürich, Switzerland

**Keywords:** Pearson correlation, current transformer, temperature, frequency, partial correlation

## Abstract

The article elaborates on the accuracy of current transformers (CT) in interaction with temperature and frequency using Pearson’s correlation. The first part of the analysis compares the accuracy of the mathematical model of the current transformer and the result of the measurement on the real CT using the Pearson correlation calculation. The mathematical model of CT is determined by deriving the formula of the functional error with the display of the accuracy of the measured value. The accuracy of the mathematical model is affected by the accuracy of current transformer model parameters and the calibration characteristic of the ammeter used to measure the CT current. Variables that cause deviation in the accuracy of CT are temperature and frequency. The calculation shows the effects on accuracy in both cases. The second part of the analysis refers to the calculation of the partial correlation of three quantities: (1) CT accuracy, (2) temperature, and (3) frequency on a set of 160 measurements. First, the influence of temperature on the correlation of CT accuracy and frequency is proven, following the proof of the influence of frequency on the correlation of CT accuracy and temperature. In the end, the analysis is combined by comparing the measured results of the first and second part of the analysis.

## 1. Introduction 

This paper considers the process system identification methodology applied to the current transformer [1]. The theory of identification of process systems uses two basic methods: non-parametric and parametric identification procedures [2].

- Non-parametric methods are performed in the time domain. Fourier analysis and correlation analysis are used in that case.
- Identification using the parameter estimation procedure uses static variables. This method is characterized by the need to evaluate the initial model, which must be performed in order to obtain convergence towards the final solution. The process is, finally, described by a transfer function.

The specificity of the experiment is the use of mathematical procedures of Pearson correlation of measured and calculated values to obtain the most accurate initial model possible. The analysis highlights anomalies detected during statistical analysis of input and output quantities. The partial correlation calculation includes the third quantities as functions of disturbance, namely temperature and frequency.

The accuracy of measuring transformers is defined by accuracy class, transmission ratio, and rated power. These are numerical quantities. The error of the measuring transformer is a variable quantity that depends on the secondary load. The class index represents the maximum allowable error of the instrument when used in reference conditions. The error limits can be expressed in percentages of the scale length, the true value, or, most often, in percentages of the maximum value of the measuring range. The accuracy classes of current transformers are 0.1; 0.2; 0.5; 1; 3; and 5. Some omissions and approximations were made in the derived model. Physical phenomena such as hysteresis losses and eddy current losses are neglected in the model due to the small estimated amount. Frequency and temperature affect the amounts of core and winding losses. This is the reason for using the Pearson correlation as a statistical method [3,4]. As a result of the analysis, partial correlation factors are presented in the article as evidence of the interaction of three variables: CT accuracy, frequency [5,6], and temperature. The correction amounts obtained by partial correlation can be used as a justification for neglecting these influences, depending on the accuracy requirements [7].

## 2. Methodology of CT Accuracy Analysis Using Pearson Correlation Factors and Regression Factors

In the analysis of the measured data, we will use the regression factor “*r*^2^” and the correlation factor “*r*”, terms used in statistics and error theory. There is an interrelation between the regression factor “*r*^2^” and the correlation “*r*”, and the definitions of these two quantities should be distinguished because these values are related, but their interpretations are different:

- The amount “*r*^2^” indicates how well the regression line approximates a certain set. It is a percentage value (%) that determines how much of the observed set is within the given variation. It ranges from 0 to 1, i.e., from 0 to 100%.
- The correlation factor “*r*” is used when one wants to prove the relationship between two variables and the strength of that relationship. It is used with a set of two quantities and ranges from −1 to +1.

The analysis of the two quantities e_m_ and e_f_, i.e., the functional error of the CT and the measured error of the CT, are shown below. Those two quantities are dependent variables that depend on the percentage (%) of CT load. If we consider them as variables that describe the real and ideal measuring system, we can write them as dependent variables on the ordinate axis and the abscissa axis. They are not equal, but they are similar in terms of amounts, and we assume their connection, which we will try to prove statistically.

**Figure 1:**
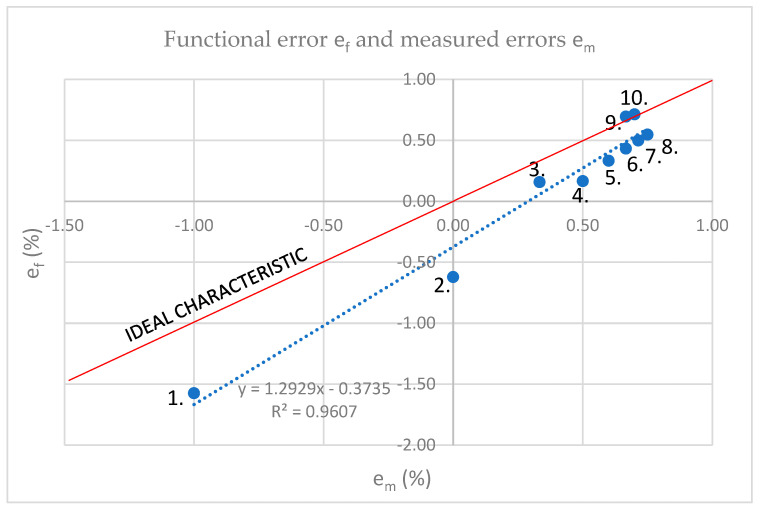
Ideal characteristic, measured point and regression line of CT at *f =* 100 Hz, *T =* 26.3 °C.

The goal of the analysis is to detect deviations and anomalies in the entire measurement area of the CT and to prove it by measurement. The diagram in Figure 1 graphically interprets data listed in Table 1:

The ideal characteristic represents the direction in which real measurements match the calculation model, i.e., functional amounts, 100%. The closer the real measurements are to the ideal characteristic cause the higher the quality of the computational model. Measurements are performed at a frequency of 100 Hz.

The formula of the measured characteristic is:

(1) $$\;\hat{y}=b_{0}+b_{1}\cdot x=-0.373+1.292\cdot x$$

*b*_0_ = −0.373; *b*_1_ = 1.292 (2)

The formula of the ideal characteristics is:

(3) $$\hat{y}=b_{0}+b_{1}\cdot x=x$$

*b*_0_ = 0; *b*_1_ = 1 (4)

It can be seen that the ideal characteristic, *b*_1_ = 1, which, considering Formula (3), means that the correlation must be *r* = 1, and the standard deviations s_x_ and s_y_ must have the same value. For the real characteristic, some deviation results are shown in Formula (1).

### Trajectory Obtained Using “Spline” Interpolation

**Figure 2:**
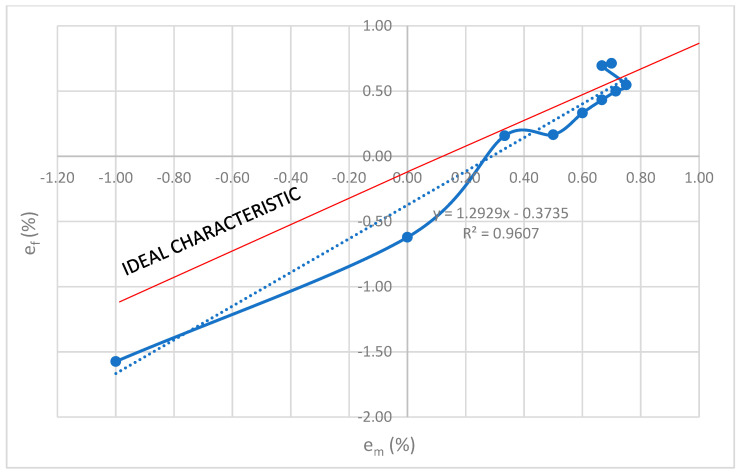
Interpolation “spline” curve in statistical experiment.

A specific curve in Figure 2 is obtained by connecting the points by “spline” type interpolation in the order in Table 1. The independent variable is the primary CT current, which increases linearly from 0 to 100% with a step of 10%. Two functions with dependent variables e_m_ and e_f_ were used to graphically display the measure of deviation from the ideal characteristic, one on the ordinate axis and the other on the abscissa axis. This way of displaying results was applied in the rest of the work to all experiments.

The benefit of “spline” interpolation:

- visualization of the measurement characteristics of the current transformer
- display of deviations on the entire measuring area
- determination of the place on the curve that contributes to the deterioration of the correlation factor
- insight into the sensitivity of trajectory
- insight into the slope of the trajectory

**Figure 3:**
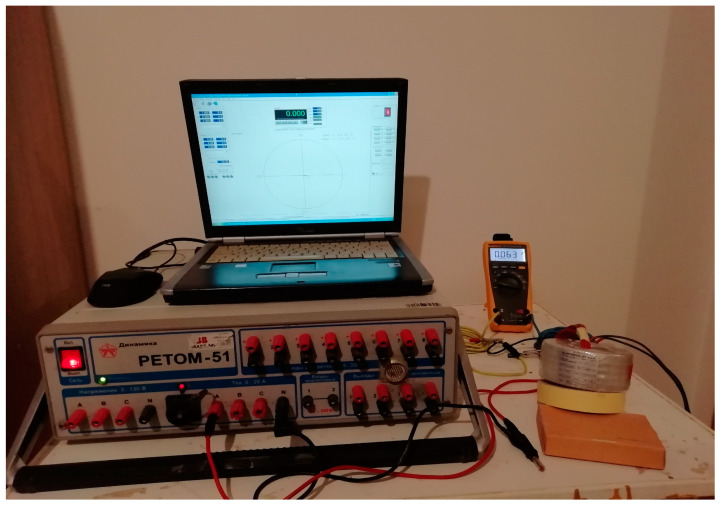
Setting of the experiment.

Figure 3 displays the instruments used for this experiment. The parts used for the test are personal computer, current injection device, current transformer, and high precision measuring ammeter.

## 3. Pearson Correlation—Application to the Test

Correlation represents the mutual relationship between different sets of data represented by variables. Data sets can be stochastic or determined by a function. In this case, these are the percentage amounts of two errors: complex function errors e_f_ and measured CT errors e_m_.

The correlation coefficient is determined by the Formula (5). The value of the correlation coefficient ranges from “+1” to “−1”, i.e., from perfect positive correlation to perfect negative correlation. The correlation coefficient is based on the comparison of the interaction of two variables in relation to the maximum possible influence of the two variables. This correlation coefficient is also called the Pearson correlation coefficient [3,4].

(5) $$r=\frac{\sum (X_{i}-\bar{X})\left(Y_{i}-\bar{Y}\right)}{\sqrt{\sum_{i}^{n}{\left(X_{i}-\bar{X}\right)}^{2}+\sum_{i}^{n}{\left(Y_{i}-\bar{Y}\right)}^{2}}}$$

The use of correlation in measurement accuracy should be carried out with some limitations. The paper considers the relationship between function points. These function points can be declared as a set of stochastic points, so the correlation of these two sets of declared stochastic points is calculated according to the Formula (5).

Correlation does not determine measurement accuracy and error limits, which is shown in the following example.

Task: Calculate the Pearson correlation coefficient between two hyperbolas defined by the equations *y*_1_(*x*) = *a*/*x* + *b* and *y*_2_(*x*) = *c/x* + *d* on a limited interval. 

Excerpt:

Two following equations:

(6) $$y_{1}\left(x\right)=a\cdot f\left(x\right)+b$$

(7) $$y_{2}\left(x\right)=c\cdot f\left(x\right)+d$$

are equated by using the common function *f*(*x*). It follows:

(8) $$\frac{y_{1}\left(x\right)-b}{a}=\frac{y_{2}\left(x\right)-d}{c}\;$$

(9) $$y_{1}\left(x\right)=y_{2}\left(x\right)\;\frac{a}{c}\;+\left(b-\frac{da}{c}\right)$$

A linear dependence of *y*_1_(*x*) and *y*_2_(*x*) was obtained, which represents the direction of regression and assumes a perfect correlation of “+1” if the coefficient *a/c* is positive. Otherwise, the correlation is “−1”. Accordingly, two functions with different amounts can have a correlation coefficient of +1 i.e., perfect correlation.

The reverse is also valid: if two different functions *y*_1_(*x*) and *y*_2_(*x*) have a “+1” correlation, then they can be written in the form of linear equations and have a common function *f*(*x*).

We can conclude that the correlation is perfect, i.e., equal to “+1” for the entire family of curves:

(10) $$y\left(x\right)=\frac{a}{x}+b$$

## 4. Functional Error Analysis of the Current Transformer Transfer Ratio

**Figure 4:**
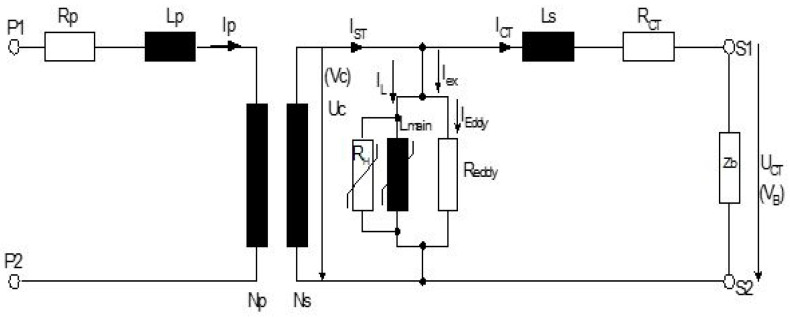
Equivalent circuit of the current transformer.

The replacement model of the current transformer is shown in Figure 4. Table 2 gives the factory testing results of current transformer.

**Figure 5:**
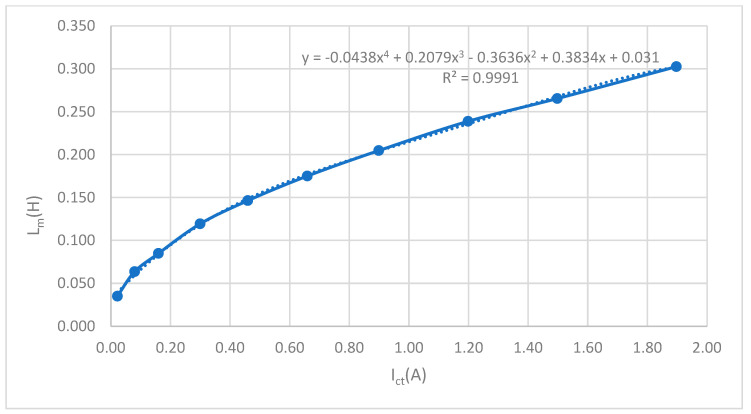
Dependance of the inductance *L_m_* on the secondary current of CT.

The variable transformation procedure can be seen in Table 3. The result is the curve *L_m_* = *f* (*I_ct_*) given in Figure 5 defined by Formula (11) obtained by the interpolation of points in the transformation in Table 3. This equation will be needed to determine *L_m_* as a function of current. It should be pointed out that CT is analyzed with external load *Z_b_* = 0. Information about the protective current transformer is given in Table 2. The material used for magnetic core was “Grain Oriented Silicon Steel Strips; Grade VM 97–27”.

**Figure 6:**
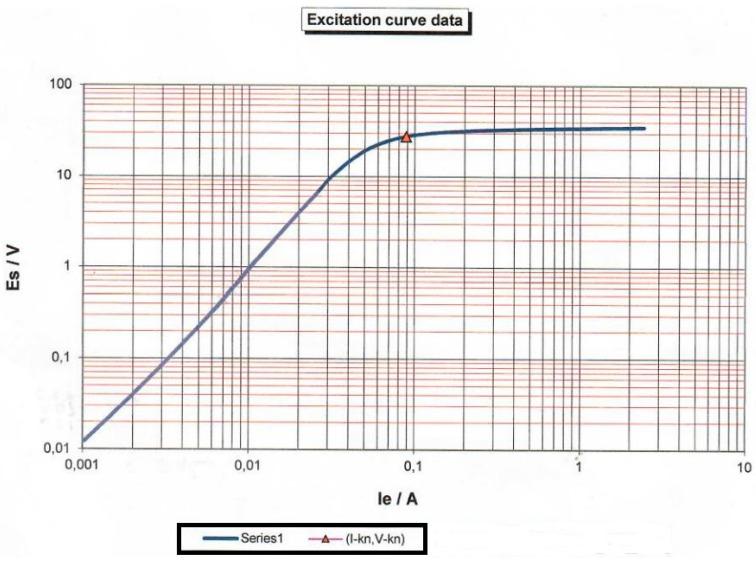
Test result of CT magnetization.

Figure 6 is connected with Table 3. Variables *E_s_* and *I_e_* are manually inserted in Table 3. Figure 6 and Table 3 were necessary to obtain Formula (11), which will be used for calculation functional error of CT (27).

A curve is obtained by the inclusion *L_m_ = f*(*I_ct_*): 

Following the formula obtained using the 4th degree polynomial approximation from the data processing program:

*L_m_* = −0.0438*·I_ct_*^4^ + 0.2079·*I_ct_*^3^ − 0.3636*·I_ct_*^2^ + 0.3834*·I_ct_* + 0.031 (11)

## 5. Derivation of the Current Transformer Functional Error

**Figure 7:**
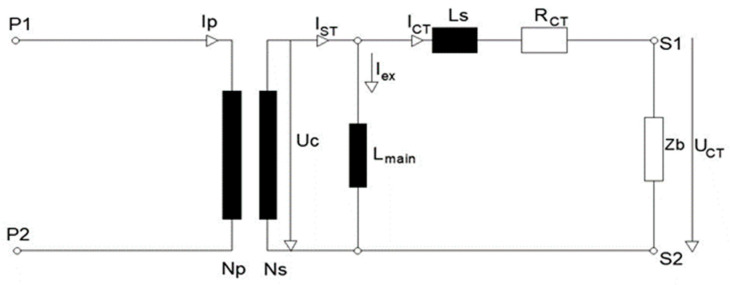
Reduced model of current transformer.

This part of the analysis refers to the determination of the functional fault on the secondary current fault. The amounts of resistance *R_h_* and *R_eddy_* in Figure 1 are many times higher than the inductive resistance *X_L_ = jωL_m_* and can be treated as infinitely large, i.e., ignored. The reduced model of the current transformer for *R_h_>>* and *R_eddy_>>* is shown in Figure 7:

Below is a derivation of the error function of an unloaded current transformer with a short-circuited secondary circuit:

(12) $$e=\frac{I_{ct}-I_{st}}{I_{st}}\cdot$$

It follows:

(13) $$\frac{I_{ex}}{I_{ct}}=\frac{Z_{ct}}{Z_{ex}}\;\;$$

(14) $$I_{ct}=I_{st}-I_{ex}=I_{st}-I_{ct}\frac{Z_{ct}}{Z_{ex}}$$

(15) $$Z_{ex}=j\cdot \omega L_{m};\;Z_{ct}=R_{ct}+j\cdot \omega L_{s}$$

(16) $$I_{ct}=I_{st}\frac{Z_{ex}}{Z_{ex}+Z_{ct}}=I_{st}\frac{j\omega L_{m}}{j(\omega L_{m}+\omega L_{s})+R_{ct}}$$

(17) $$I_{ct}=I_{st}\frac{\;j\cdot \omega \;L_{m}R_{ct}+\omega^{2}L_{m}^{2}+\omega^{2}L_{m}L_{s}}{R_{ct}^{2}+\omega^{2}(L_{m}+L_{s}){}^{2}\;}$$

(18) $$I_{ct}=I_{st}\left[\;\;j\cdot \frac{\;\omega \;L_{m}R_{ct}}{R_{ct}^{2}+\omega^{2}(L_{m}+L_{s}){}^{2}\;}+\frac{\;\omega^{2}L_{m}^{2}+\omega^{2}L_{m}L_{s}}{R_{ct}^{2}+\omega^{2}(L_{m}+L_{s}){}^{2}\;}\right]$$

(19) $$I_{ct}=I_{st}\sqrt{{\left[\frac{\;\omega \;L_{m}R_{ct}}{R_{ct}^{2}+\omega^{2}(L_{m}+L_{s}){}^{2}\;}\right]}^{2}+{\left[\frac{\;\omega^{2}L_{m}^{2}+\omega^{2}L_{m}L_{s}}{R_{ct}^{2}+\omega^{2}(L_{m}+L_{s}){}^{2}\;}\right]}^{2}}$$

The following is the expression for the amplitude of the functional error of the measurement of a current transformer with a short-circuited secondary, i.e., for *Z_b_* = 0:

(20) $$e=\left(\frac{I_{ct}-I_{st}}{I_{st}}\;\right)\cdot 100\%=\left(\frac{I_{ct}}{I_{st}}-1\;\;\right)\cdot 100\%$$

(21) $$e=\left[\sqrt{{\left[\frac{\;\omega \;L_{m}R_{ct}}{R_{ct}^{2}+\omega^{2}(L_{m}+L_{s}){}^{2}\;}\right]}^{2}+{\left[\frac{\;\omega^{2}L_{m}^{2}+\omega^{2}L_{m}L_{s}}{R_{ct}^{2}+\omega^{2}(L_{m}+L_{s}){}^{2}\;}\right]}^{2}}-1\right]\cdot 100\%$$

With:

- *L_m_* = secondary inductance *L_main_* of the current transformer, dependence on the current is given by (1)
- *ω* = circular frequency
- *R_ct_* = ohmic resistance of the secondary
- *L_s_* = inductance of the secondary connection lines

**Figure 8:**
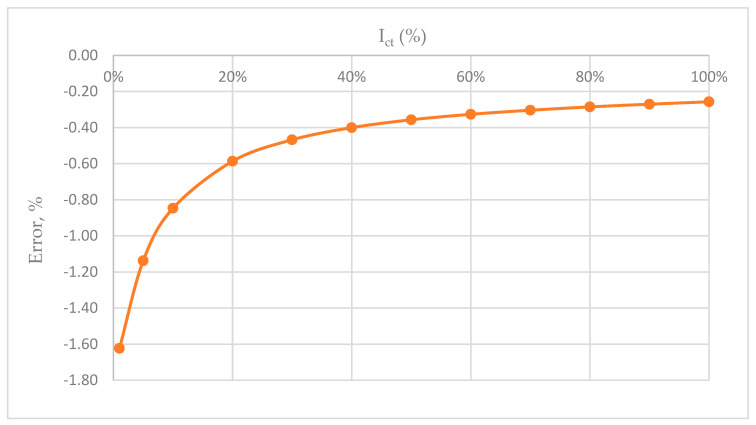
CT error function diagram.

Based on the previous formula, the display of the CT error for the frequency of 50 Hz follows. The largest amount in Figure 8 is −1.6%. We notice that the error function is shifted towards the negative part of the scale.

Manufacturers of current transformers raise the error diagram of CT for the desired value by correcting the number of windings due to the requirement for better accuracy.

## 6. Correction of the Number of Secondary Windings

**Figure 9:**
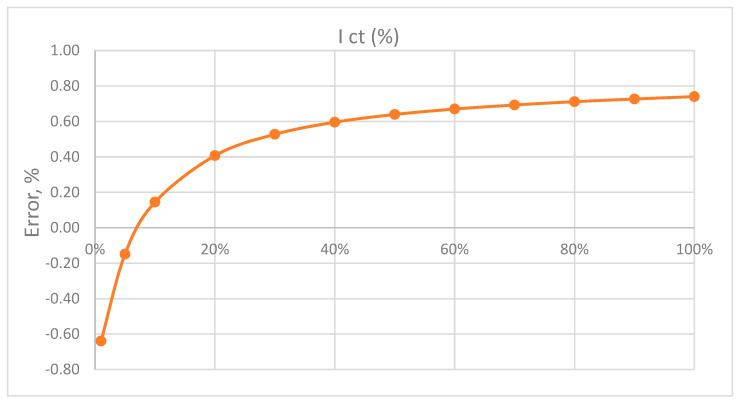
Figure of the error function with the correction of the number of windings n_k_ = 99.

By correcting the number of secondary windings, the curve in Figure 9 is raised, the maximum error on the interval (0–100%) of *I_n_* is reduced, and the expression for the new corrected secondary current is obtained:

(22) $$I_{ST}^{\prime }=I_{ST}\frac{n}{n_{k}}$$

(23) $$e=\frac{I_{\mathrm{ct}}-I_{\mathrm{st}}^{\prime }}{I_{\mathrm{st}}^{\prime }}\cdot 100\%=\left(\frac{n\cdot I_{\mathrm{ct}}}{n_{k}\cdot I_{\mathrm{st}}}-1\right)\cdot 100\%$$

Inserting the following expression for the CT error:

(24) $$e=\left[\frac{n}{n_{k}}\cdot \sqrt{{\left[\frac{\;\omega \;L_{m}R_{ct}}{R_{ct}^{2}+\omega^{2}(L_{m}+L_{s}){}^{2}\;}\right]}^{2}+{\left[\frac{\;\omega^{2}L_{m}^{2}+\omega^{2}L_{m}L_{s}}{R_{ct}^{2}+\omega^{2}(L_{m}+L_{s}){}^{2}\;}\right]}^{2}}-1\right]\cdot 100\%$$

With:

- *L_m_* = secondary inductance *L_main_* of the current transformer, and dependence on the current is given by (11)
- *ω* = circular frequency
- *R_ct_* = ohmic resistance of the secondary
- *L_s_* = inductance of the secondary connection lines
- *n_k_* = corrected number of windings
- *n* = number of secondary windings

**Figure 10:**
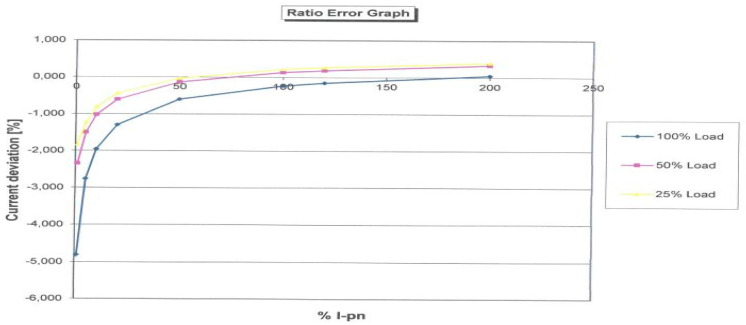
Factory testing results of ratio error.

Figure 9 shows the CT diagram with the number of corrected windings. The number of windings was reduced from 100 to 99. This reduces the maximum measurement error for nominal values to 0.74%, and the interval within which the error is located is symmetrical around the abscissa of axis X. The comparation can be performed with a factory testing result of CT, which is displayed in Figure 10 for three specific loads.

## 7. Calibration of the Measuring Instrument

The measuring instrument can cause inaccuracy of the measurement result, so it is necessary to calibrate it. The CT current measurement system consists of a certified current source, a *k* = 100/1 transmission ratio current transformer, a class 10P10, and a measuring instrument, Fluke 86. It follows that:

- *A*; constant current source (Amp)
- *B* = (*A/k*) · (1 − *ε*_1_); “ε_1_” is functional error of the current transformer, “*B*” is secondary current of CT, “*k*” is current ratio
- *C = B ·* (1 *− ε*_2_); “ε_2_” measuring instrument error, “*C*” is current displayed on ammeter

By including the above we obtain:

- *C* = (*A/k*) · (1 *− ε*_1_) · (1 − *ε*_2_) = (*A/k*) · (1 − *ε*_1_ − *ε*_2_ + *ε*_1_ · *ε*_2_) (*Amp*)

Follows a secondary current of CT corrected by the complex measurement error:

*C* ≈ (*A/k*) · (1 *− ε*_1_
*− ε*_2_) (25)

with the assumption *ε*_1_, *ε*_2_ <<, *C* is measured secondary current. 

**Figure 11:**
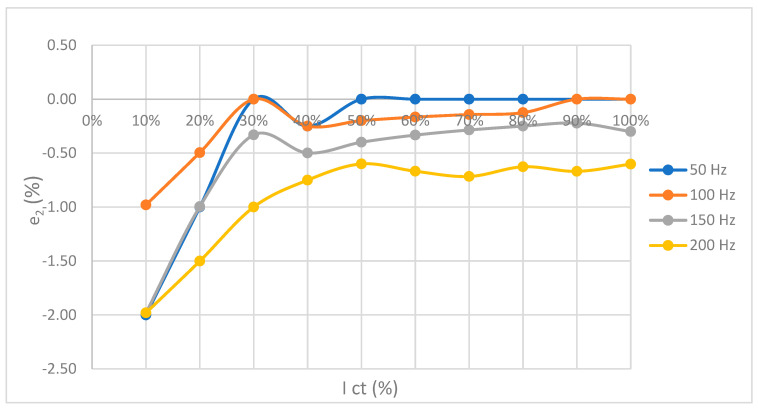
Display of the ammeter calibration curve at a temperature of 26.3 °C.

The Fluke 86 ammeter was calibrated for the specified current and frequency. The goal was to obtain the complex error of the measuring system. This error consists of the functional error of the current transformer and the calibration error of the measuring device. Figure 11 shows a set of calibration curves for four frequencies 50 Hz, 100 Hz, 150 Hz, and 200 Hz measured at room temperature 26.3 °C.

## 8. Functional Dependence of CT Measurement Error on Temperature

By including the temperature in the expression for the functional error (24) of the current transformer, the error values are obtained depending on the temperature. The formula for the temperature coefficient of electrical resistance is:

*R_ct_*(*T*) = *R*_0_ · [1 + *α* · (*T* − *T*_0_)] (26)

with:

- *α* = temperature coefficient of electrical resistance, *α* = 0.00386 1/*K*
- *T*_0_ = initial temperature, 20 °C
- *R*_0_ = electrical resistance at temperature *T*_0_, *R*_0_ = 0.445 Ω

In the formula for the CT error (27), the resistance is predicted to change in the variable *R_ct_*, so the temperature values for currents from 0.1 A to 1 A are included. The expression for the error is defined as follows:

(27) $$e=[\frac{n}{n_{k}}\cdot \sqrt{{\left[\frac{\;\omega \;L_{m}R_{ct}}{R_{ct}^{2}+\omega^{2}(L_{m}+L_{s}){}^{2}\;}\right]}^{2}+{\left[\frac{\;\omega^{2}L_{m}^{2}+\omega^{2}L_{m}L_{s}}{R_{ct}^{2}+\omega^{2}(L_{m}+L_{s}){}^{2}\;}\right]}^{2}}-1]\cdot 100\%$$

## 9. Temperature 26.3 °C—Results

**Figure 12:**
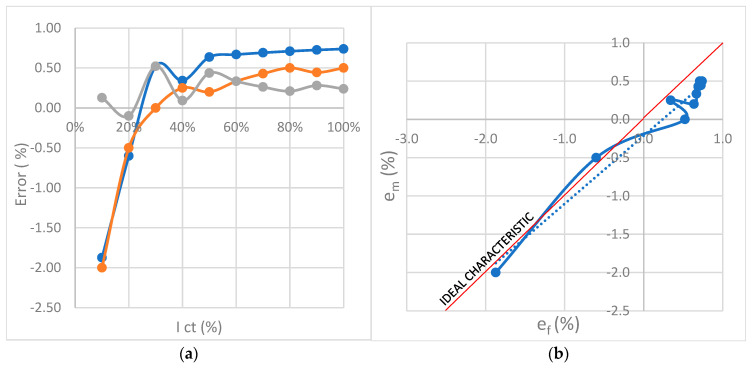
CT error depending on the secondary current for a temperature of 26.3 °C, *f* = 50 Hz. (**a**) measured (red); function (blue); difference (gray). (**b**) “Spline” curve of function error e_f_ and measured error e_m_; *R*^2^ = 0.9624.

Figure 12 shows the diagrams of complex function error, measurement error, and error difference.

## 10. Functional Dependence of CT Measurement Error on Frequency

In the formula for CT error (28), the frequency is predicted to change in the variable w, so the frequency values are 50 Hz to 200 Hz. The current range ranges from 0.1 A to 1 A. The expression for the error is, in this case [5,6]:

(28) $$e=[\frac{n}{n_{k}}\cdot \sqrt{{\left[\frac{\;\omega \;L_{m}R_{ct}}{R_{ct}^{2}+\omega^{2}(L_{m}+L_{s}){}^{2}\;}\right]}^{2}+{\left[\frac{\;\omega^{2}L_{m}^{2}+\omega^{2}L_{m}L_{s}}{R_{ct}^{2}+\omega^{2}(L_{m}+L_{s}){}^{2}\;}\right]}^{2}}-1]\cdot 100\%$$

By correcting the number of windings, a symmetrical distribution of the curves around the abscissa axis X was obtained. The error e_hz_ ranges from −0.65% to 0.74%. 

### Frequency 50, 100, 150, 200 Hz—Results

**Figure 13:**
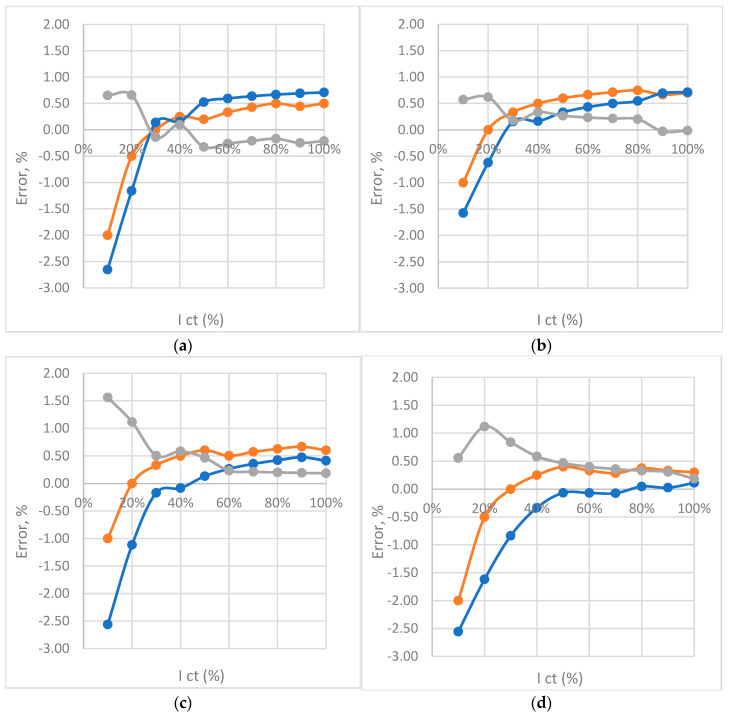
CT error curves depending on the secondary current for the frequency: (**a**) *f* = 50 Hz measured (red); function (blue); difference (gray). (**b**) *f* = 100 Hz measured (red); function (blue); difference (gray). (**c**) *f* = 150 Hz measured (red); function (blue); difference (gray). (**d**) *f* = 200 Hz measured (red); function (blue); difference (gray).

Follows a presentation of the obtained results for frequencies 50 Hz, 100 Hz, 150 Hz, and 200 Hz. The values obtained from the model are compared with the measured values, and the data defining the accuracy and quality of the model parameters are shown. Figure 13 shows the diagrams of complex function error, measurement error, and error difference.

**Figure 14:**
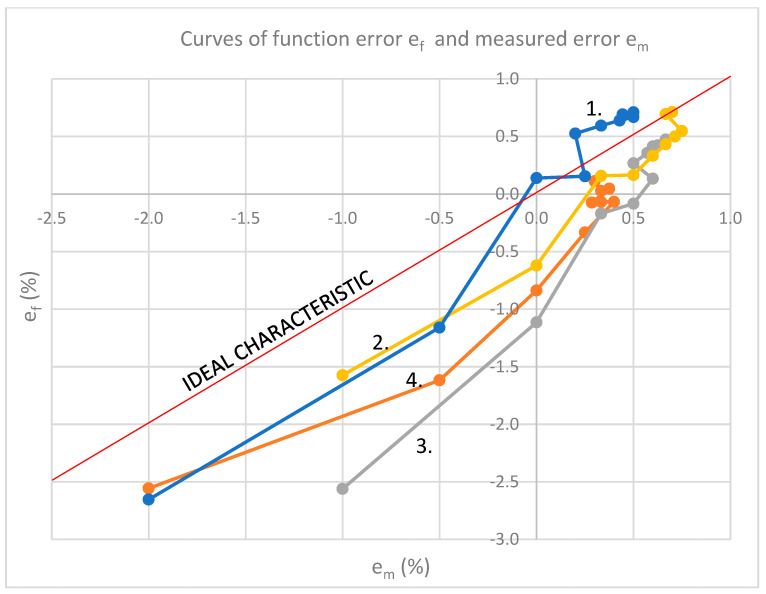
Curve: **1.** *f* = 50 Hz; *R*^2^ = 0.9657, **2**. *f* = 100 Hz; *R*^2^ = 0.9607, **3**. *f* = 150 Hz; *R*^2^ = 0.9727, **4**. *f* = 200 Hz; *R*^2^ = 0.916.

Figure 14 represents four curves of function error e_f_ and measured error e_m_. Differences between curves can be seen depending on frequency [8,9].

## 11. Discussion about Results of Measurements

The curves from Figure 14 are divided into 9 segments shown in Table 4 using the result of derivation (12), the analysis of the obtained results was performed. Each segment is correlated with an ideal characteristic. The sign of the curve slope is marked with + or −. It determines the correlation of the segment of the measured curve with the segment of the ideal characteristic. Negative results are marked as “−1”. On these parts, the proposed model behaves differently from the measured model, i.e., the correlation is negative. Since the example that considers frequency dependence is taken, it is possible to refine the CT model and cancel negative correlations on all segments. The parts marked as “−1” point out the imperfection of the CT or measuring instrument, but also the shortcomings of the model. The largest correlation oscillations are at 200 Hz. 

The quality of this analysis also depends of the number of segments. The higher the number cause the more precise the segment determination. Moreover, the omissions that were made at the beginning should not be ignored and can have influence on the marked points. 

## 12. Partial Correlation of Temperature, Frequency, and CT Measurement Error

If we calculate the connection between two phenomena, it is sometimes necessary to exclude the importance of a third variable that can affect the amount of connection between the first two variables. Partial correlation shows the correlation between two variables where the influence of the third variable is excluded. It is calculated according to the formula [4]:

(29) $$r_{12/3}=\frac{r_{12}-r_{13}\cdot r_{23}}{\sqrt{1-r_{13}^{2}}\;\cdot \;\sqrt{1-r_{23}^{2}}}$$

- *r*_12_–correlation coefficient between the 1st and 2nd variables; *r*_13_–correlation coefficient between the 1st i 3rd variables; *r*_23_–correlation coefficient between the 2nd and 3rd variables

The correlation coefficient is defined by the formula:

(30) $$r_{xy}=\frac{\sum (X_{i}-\bar{X})\left(Y_{i}-\bar{Y}\right)}{\sqrt{\sum_{i}^{n}{\left(X_{i}-\bar{X}\right)}^{2}+\sum_{i}^{n}{\left(Y_{i}-\bar{Y}\right)}^{2}}}$$

In the analysis of the influence of temperature and frequency on the accuracy of the current transformer, we will use the measurement results. Ten measurements were performed for four frequencies and four temperatures, for a total of 4 × 4 × 10 = 160 measurements. In Section 8, the functional dependence of the CT error on temperature was shown, and in Section 10, the functional dependence of the CT error on frequency was shown. 

A prerequisite for the calculation of the partial correlation is the calculation of the matrix of all correlations, which is given in Table 5.

- *r*_12_–correlation coefficient between temperature and frequency, *r*_12_ = 0
- *r*_13_–correlation coefficient between temperature and CT error, *r*_13_ = −0.3137
- *r*_23_–correlation coefficient between frequency and error CT-a, *r*_23_ = −0.0137

When calculating partial correlation, we obtain the “correct” correlation between the quantities, which, in the specific case, means:

- The influence of frequency on the relationship between temperature and error is *r*_13/2_ = −0.3138 (Table 6), which is very close to the correlation of these two quantities, *r*_13_ = −0.3137 (Table 5). The conclusion is that the frequency does not influence the relationship between CT error and measurement temperature.
- The influence of temperature on the frequency and CT error is *r*_23/1_ = −0.0144 (Table 6), which is very close to the linear correlation of these two quantities, *r*_13_ = −0.0137 (Table 5). The conclusion is that temperature has no significant influence on the relationship between CT error and measurement frequency.

It should be noted that the partial correlation *r*_12/3_ was not considered due to the illogicality of the results of that measurement.

- *r*_12/3_–partial correlation coefficient between temperature and frequency, *r*_12/3_ = 0
- *r*_13/2_–partial correlation coefficient between temperature and CT error, *r*_13/2_ = −0.3137
- *r*_23/1_–partial correlation coefficient between frequency and CT error, *r*_23/1_ = −0.0137

At the end, Table 7 displays the changes after the correction of correlation and eliminates influences of the frequency (the change: −0.003%) and temperature (the change: −5.1%).

## 13. Conclusions

As is pointed out in Section 11, the Pearson correlation can be used to improve the model of CT. Using the proposed model, we can define the point where the deviation from the model is significant. There are two ways to be near to the ideal characteristic: To correct the mathematical model or to change the performance of CT. The Pearson correlation in this work shows possible errors which are recognized as changes of slope in comparison with the ideal characteristic. 

Generally, the deviation of the measured and calculated value shown in Figure 13b and Figure 14a–d was the impetus for determining the partial correlation of temperature and frequency to the measurement error. The regression factor, same as Pearson correlation, has high values. The calculation proved that there is no significant influence of each of these quantities on the interconnection of another quantity with measurement error, except in the cases pointed out in Section 11. 

Moreover, the influence of the digital measuring instrument is the limiting factor. The measurement limit of three decimal places can disturb the total result and bring in a disturbance.

At the end of experiment, the calculation of partial correlation between three factors is performed: temperature, frequency, and function of error. This verification is useful to estimate the influence of external variables such as temperature and frequency, to be sure they have an influence on the mutual interference of the two monitored variables. 

The whole experiment can be considered successful from the perspective of developing a method of determining the parameters of the current transformer using a statistical method [10,11].

## Figures and Tables

**Table 1:** Example of functional and measured error for *f* = 100 Hz.

No.	1.	2.	3.
	I (%)	e_m_ (%)	e_f_ (%)
1.	10%	−1.000	−1.574
2.	20%	0.000	−0.620
3.	30%	0.333	0.157
4.	40%	0.500	0.165
5.	50%	0.600	0.332
6.	60%	0.667	0.432
7.	70%	0.714	0.499
8.	80%	0.750	0.547
9.	90%	0.667	0.694
10.	100%	0.700	0.713
st. dev. s		**s** **_x_** ** = 0.5126**	**s** **_y_** ** = 0.6761**

**Table 2:** Results of factory test of current transformer.

Nr.	Test Settings		Nr.	Excitation Test		Nr.	Ratio Test	
1	I-pn	100 A	14	V-kn	27.663 V	27	Ratio	100:0.99766
2	I-sn	1 A	15	V-kn 2	n.a.	28	e	−0.2341%
3	Rated burden	2.5 VA/1	16	Ls	0.0005523 H	29	e_c_	1.2535%
4	Operating burden	2.5 VA/1	17	Kr	89.07%	30	Df	41.82 min
5	Applied standard	IEC 61869-2	18	I-kn	0.088602	31	Polarity	ok
6	Core type (P/M)	P	19	I-kn 2	n.a.	32	N	99
7	Class	10 P	20	Lm	1.2104 H			
8	ALF	10.0						
9	f	50.0 Hz	nr.	Result with rated burden				
10	Ts	n.a.	21	ALF	11.63			
11	max. R_ct_	0.531 W	22	Ts	0.399 s			
			23	ALFi	11.4			
nr.	Resistance test							
12	R_meas_ (25 °C)	0.44532 W	nr.	Result with operating burden				
13	R_ref_ (75 °C)	0.53112 W	24	ALF	11.63			
			25	Ts	0.399 s			
			26	ALFi	11.4			

**Table 3:** Factory data of the saturation magnetic curve.

*I_e_* (A)	0.001	0.002	0.003	0.004	0.005	0.006	0.007	0.008	0.009	0.01
*E_s_* (V)	0.011	0.04	0.08	0.15	0.23	0.33	0.45	0.6	0.75	0.95
*L_m_* = *E_s_*/(*I_e_*·314) (H)	0.035	0.064	0.085	0.119	0.146	0.175	0.205	0.239	0.265	0.303
|*R_ct_* + *jwL_s_*| (Ω)	0.50	0.50	0.50	0.50	0.50	0.50	0.50	0.50	0.50	0.50
*I_ct_* = *E_s_*/|*R_ct_* + *jwL_s_*| (A)	0.02	0.08	0.16	0.30	0.46	0.66	0.90	1.20	1.50	1.90

**Table 4:** Correlation of ideal curve segments and measured curve segments.

Point	1. Segment	2. I (%)	3. Ideal Curve SlopeSign	4. Curve Slope Sign at 50 Hz/ Correlation 3. and 4.	5. Curve Slope Sign at 100 Hz/ Correlation 3. and 5.	6. Curve Slope Sign at 150 Hz/ Correlation 3. and 6.	7. Curve Slope Sign at 200 Hz/ Correlation 3. and 7.
1.		10%					
2.	1–2	20%	+	+/1	+/1	+/1	+/1
3.	2–3	30%	+	+/1	+/1	+/1	+/1
4.	3–4	40%	+	+/1	+/1	+/1	+/1
5.	4–5	50%	+	−/−1	+/1	+/1	+/1
6.	5–6	60%	+	+/1	+/1	−/−1	−/−1
7.	6–7	70%	+	+/1	+/1	+/1	−/−1
8.	7–8	80%	+	+/1	+/1	+/1	+/1
9.	8–9	90%	+	−/−1	−/−1	+/1	−/−1
10.	9–10	100%	+	+/1	+/1	+/1	−/−1
	PEARSON CORRELATION “e_m_ ∧ e_f_”			0.9827	0.98	0.9862	0.957

**Table 5:** Correlation matrix.

	Temp.; R_1_	Freq.; R_2_	Error CT; R_3_
**Temp.; R_1_**	1.0000	0.0000	−0.3137
**Freq.; R_2_**	0.0000	1.0000	−0.0137
**Error CT; R_3_**	−0.3137	−0.0137	1.0000

**Table 6:** Matrix of partial correlation–influence on connection.

	Freq.; R_2_	CT Error; R_3_	Temp.; R_1_
**Temp/CT Error; R_13_**	−0.3138		
**Temp/Freq; R_12_**		−0.0045	
**Freq./CT Error; R_23_**			−0.0144

**Table 7:** Relations of values.

Correlation	Value Before Correction	Value After Correction	D%
**1. Temp./CT Error**	−0.3137	−0.3138	−0.003%
**2. Freq./CT Error**	−0.0137	−0.0144	−5.1%

## References

[1] (2018). CT-Analyzer-Test Ceritficate nr. 111/12/C.

[2] Perić N., Petrović I. (2002). Identifikacija Procesa—Predavanja.

[3] Galić R. (1999). Vjerojatnost i Statistika.

[4] Luchka A.Y. (1963). The Method of Averaging Functional Correction.

[5] Saleh S.M., El-Hoshy S.H., Gouda O.E. (2017). Proposed diagnostic methodology using the cross-correlation coefficient factor technique for power transformer fault identification. IET Electric Power Applications.

[6] Behjat V., Mahvi M., Rahimpour E. (2016). New statistical approach to interpret power transformer frequency response analysis: Non-parametric statistical methods. IET Science, Measurement & Technology.

[7] Herceg D. (1989). Numeričke i Statističke Metode u Obradi Eksperimentalnih Podataka.

[8] Yachikov I.M., Nikolaev A.A., Zhuravlev P.Y., Karandaeva O.I. (2017). Estimate of Correlation between Transformer Diagnostic Variables with a Time Lag. Int. Conf. Ind. Eng. ICIE.

[9] Wang X., Li Q., Yang R., Li C., Zhang Y. (2014). Diagnosis of solid insulation deterioration for power transformers with dissolved gas analysis-based time series correlation. IET Sci. Meas. Technol..

[10] Guillen D., Esponda H., Vasquez E., Idarraga-Ospina G. (2016). Idarraga-Ospina. Algorithm for transformer differential protection based on wavelet correlation modes. IET Generation, Transmission & Distribution.

[11] Yu T., Yang J., Lin J.C., Geng C. (2020). Correlation Analysis of Transformer Parameters Based on Pair-Copula. IOP Conf. Ser. Earth Environ. Sci..

